# Complement C3 Produced by Macrophages Promotes Renal Fibrosis via IL-17A Secretion

**DOI:** 10.3389/fimmu.2018.02385

**Published:** 2018-10-22

**Authors:** Yanyan Liu, Kun Wang, Xinjun Liang, Yueqiang Li, Ying Zhang, Chunxiu Zhang, Haotian Wei, Ran Luo, Shuwang Ge, Gang Xu

**Affiliations:** ^1^Division of Internal Medicine, Department of Nephrology, Tongji Hospital, Tongji Medical College, Huazhong University of Science and Technology, Wuhan, China; ^2^Hubei Cancer Hospital, Tongji Medical College, Huazhong University of Science and Technology, Wuhan, China

**Keywords:** complement component 3, macrophage, renal fibrosis (RF), IL-17A, IgAN

## Abstract

Complement synthesis in cells of origin is strongly linked to the pathogenesis and progression of renal disease. Multiple studies have examined local C3 synthesis in renal disease and elucidated the contribution of local cellular sources, but the contribution of infiltrating inflammatory cells remains unclear. We investigate the relationships among C3, macrophages and Th17 cells, which are involved in interstitial fibrosis. Here, we report that increased local C3 expression, mainly by monocyte/macrophages, was detected in renal biopsy specimens and was correlated with the severity of renal fibrosis (RF) and indexes of renal function. In mouse models of UUO (unilateral ureteral obstruction), we found that local C3 was constitutively expressed throughout the kidney in the interstitium, from which it was released by F4/80^+^macrophages. After the depletion of macrophages using clodronate, mice lacking macrophages exhibited reductions in C3 expression and renal tubulointerstitial fibrosis. Blocking C3 expression with a C3 and C3aR inhibitor provided similar protection against renal tubulointerstitial fibrosis. These protective effects were associated with reduced pro-inflammatory cytokines, renal recruitment of inflammatory cells, and the Th17 response. *in vitro*, recombinant C3a significantly enhanced T cell proliferation and IL-17A expression, which was mediated through phosphorylation of ERK, STAT3, and STAT5 and activation of NF-kB in T cells. More importantly, blockade of C3a by a C3aR inhibitor drastically suppressed IL-17A expression in C3a-stimulated T cells. We propose that local C3 secretion by macrophages leads to IL-17A-mediated inflammatory cell infiltration into the kidney, which further drives fibrogenic responses. Our findings suggest that inhibition of the C3a/C3aR pathway is a novel therapeutic approach for obstructive nephropathy.

## Introduction

Renal fibrosis (RF) has become an important worldwide health problem and represents a major economic burden to society. IgA nephropathy (IgAN) is one of the most common causes of chronic kidney disease (CKD), and the prognosis of IgAN is more closely associated with the severity of interstitial injury and fibrosis than that of glomerular lesions (1). As the common consequence of all forms of CKD, RF is characterized by deposition of extracellular matrix and associated with inflammatory cell recruitment, angiogenesis, lymphangiogenesis, an myofibroblast formation (2–4). Although researchers have confirmed that chronic inflammation, oxidative stress, proteinuria, and abnormal activation of complement are involved in the development and progression of CKD, the pathogenesis of RF remains largely unknown (5–8).

The complement system is a crucial part of the immune system and consists of multiple categories of components. The complement component 3 (C3), a 180 kDa glycoprotein, plays a central role in activation of the complement system. Its activation is required for both classical and alternative complement activation pathways. Circulating C3 is produced by the liver, and its extrahepatic production has been observed in other specialized cells, including mast cells, fibroblasts, smooth muscle cells, and macrophages (9, 10). These cells synthesize C3, presumably through their bioactive products, and have an important role in regulating other aspects of autoimmunity, inflammation, and pathogen host defense. Studies have confirmed that locally synthesized C3 appears to have a stronger influence on rejection than circulating C3 (11). Other reports have demonstrated that the epithelial and vascular tissues at local sites of inflammation could secrete complement components (12). Despite Xavier and Cui have demonstrated that complement C3 activation and macrophage infiltration may play important roles in the progression of interstitial fibrosis in UUO mice and human hypertensive nephropathy, the specific mechanism of local synthesis in renal interstitium by immune cells has not been thoroughly investigated (13, 14).

As early as the 1970s, Pepys first discovered the interactions between complement and adaptive immunity by observing complement-depleted mice that were unable to mount potent antibody responses (15). Previous studies have found that complement C3 deficiency is associated with impaired T cell responses in several disease models, including infections, tumors, autoimmune disease, and renal transplantation (16). More recently, researchers observed that the complement components C3, fB, fD, and C5 were upregulated as regulators of T cell immunity, as well as the C3a receptor and C5a receptor. In some kidney transplant studies, the absence of C3 was correlated with defective T cell priming, reduced T cell proliferation, and cytokine production after donor-specific restimulation (17, 18). Moreover, C3 deficiency or blockade was shown to attenuate the expansion of Ag-specific CD4^+^ and CD8^+^ T cell responses to Listeria monocytogenes in mice, and the regulation of T cell functionality by C3 might not involve the C5aR signaling pathway (19).

In this study, we aimed to identify the roles of locally synthesized C3 in the development of RF in a unilateral ureteral obstruction (UUO) model and determine whether this synthesis contributes to M1 cell responses. In addition, we studied the relationships among C3, macrophages and Th17 cells, which are involved in interstitial fibrosis.

## Materials and methods

### Renal and blood samples from patients with IgAN

Between December 2016 and July 2017, patients aged 18–63 years who underwent kidney biopsy at Tongji Hospital were recruited, and renal biopsy specimens were examined retrospectively. Forty-one patients (20 men and 21 women; mean age = 38.10 ± 12.03 years) with a pathologic diagnosis of IgAN were enrolled. Their renal and/or blood samples were obtained at the time of diagnosis. Patients were excluded from this study if they met the following conditions: <18 years of age, an inability to provide informed consent, presence of active infection, and pregnancy. Our protocol was approved by the institutional review board or ethics committee at each center. Written informed consent was obtained from all patients.

### Animal model

The animals were purchased from Charles river Laboratories (Beijing, China). Unilateral ureteral obstruction (UUO) is a popular experimental model of renal injury. Mice aged 6–8 weeks were anesthetized followed by a lateral incision on the back of the mouse. Subsequently, the left ureter was exposed and tied off with two 4.0 silk suture. Sham-operated mice underwent an identical procedure but without ureteric ligation. The therapeutic experiment was performed with the Compstatin analog Cp40 (dTyr-Ile-[Cys-Val-Trp(Me)-Gln-Asp-Trp-Sar-His-Arg-Cys]-mIle-NH2;1.7kDa) which was produced by solid-phase peptide synthesis (GL Biochem Co., Ltd., Shanghai, China), and SB290157, a C3a receptor antagonist, which was purchased from Sigma-Aldrich. UUO and sham-operated mice were treated with Cp40 (1 mg/kg) via subcutaneous injection every 12 h and SB290157 (30 mg/kg) via intraperitoneal injection daily. After 7 or 14 days, the mice were sacrificed by cervical vertebra dislocation, and then, peripheral blood, spleen, and renal tissues were collected. The mouse kidneys were fixed in 4% formalin for 24 h, processed through dehydration in a graded series of alcohol and embedded in paraffin (Wuhan Goodbio Technology Co., Ltd., Wuhan, China). The remaining sample was frozen in liquid nitrogen for later use. All animal studies were performed in accordance with our university's guidelines for animal care.

### IHC and immunofluorescence

Paraffin-embedded renal sections (3 μm) were subjected to Masson's trichome staining as previously reported (20). Paraffin-embedded renal sections (4 μm) were deparaffinized in xylene and rehydrated in graded alcohol. The endogenous peroxidase activity was blocked with 3% H_2_O_2_ at room temperature for 15 min, and non-specific proteins were blocked with 10% goat serum for 30 min. Sections were then incubated overnight with antibodies against F4/80, α-SMA (Abcam, Cambridge, MA, USA), C3 (Novus, Littleton, Colorado, USA), iNOS (Santa Cruz, Dallas, Texas, USA), CD68 (Long Island Biotech, Shanghai, China), CD3, CD4, and CD8 (Thermo Scientific, Waltham, MA, USA) at 4°C, followed by incubation with an HRP-conjugated secondary antibody and subsequently visualized with diaminobenzidine substrate and hematoxylin counterstaining. For double-labeling immunofluorescence studies, following incubation with primary antibody, sections were incubated with FITC-conjugated goat anti-rabbit (1:100; Abcam) and Goat anti-rat Alexa Fluor 594 (1:100; Invitrogen Corporation, Carlsbad, CA) for 45 min at 37°C and then counterstained with DAPI (Vector Laboratories). C3 expression was quantificated by the percentages of positive area at ×400 magnification on 10 fields exclusive of blood vessels and glomerulus per section from six mice in each group. All scorings were carried out by observers blinded to the experimental groups.

### Western blotting

Protein concentrations were quantified by a BCA protein assay kit (Beyotime Institute of Biotechnology, Shanghai, China), and 20 μg of protein was used for gel loading. GAPDH primary antibody (mouse, Santa Cruz, USA) was used at a dilution of 1:3,000, and TGF-β1, collagen I, PDGFR-β, and α-SMA (Abcam, Cambridge, MA, USA) primary antibodies were used at a dilution of 1:2,000. C3 primary antibody (rabbit, Novus, USA), p-ERK, ERK, p-p65, p-STAT3, and p-STAT5 (rabbit, CST, USA) were used at a dilution of 1:1,000. iNOS primary antibody (mouse, Santa, USA) and Arginase1 (rabbit, Santa, USA) were used at a dilution of 1:200. C3aR primary antibody (mouse, Abcam, USA) was used at a dilution of 1:1,000. The secondary antibody was used at a dilution of 1:3,000. Western blotting analysis was performed as previously described (21). The signals were detected using enhanced chemiluminescence (ECL) (Amersham Pharmacia Biotech, Piscataway, NJ).

### Mouse cell preparation

C57BL/6 mice were euthanized by cervical vertebra dislocation, and then, the whole body vasculature was flushed with a 20 ml injection of fresh PBS through a cardiac puncture. Kidneys were harvested and cut into small pieces and placed in RPMI1640 medium containing 2 mg/ml collagenase IV (GIBCO) and 100 mg/ml DNase I (Roche) for 45–60 min at 37°C with intermittent agitation. After tissue disaggregation, cells were filtered through a 40 μm cell strainer (BD Falcon, Franklin Lakes, NJ). Mononuclear cells from kidneys were then washed with cold PBS, counted, and used for flow cytometry.

### PBMC isolation

Patients and normal subjects donated 5 ml of blood collected in heparinized tubes. Blood was diluted 1:1 with PBS and overlaid onto lymphocyte separation medium (TBD sciences, Tianjin, China). After centrifugation, 3 ml of the interface containing the PBMCs was collected and diluted to 6 ml with PBS, then washed twice with cold PBS and counted. The PBMCs were collected for flow cytometric analysis.

### CFSE labeling

A CFSE stock solution (5 mM) was prepared fresh by dissolving lyophilized CFSE (Sigma-Aldrich, USA) in DMSO. Splenocytes were obtained from the spleens of naïve mice, and labeled with CFSE at 5 μM in PBS for 15 min at 37°C. Excess CFSE was quenched by adding three volumes of ice-cold FBS and incubating the cells for 5 min on ice. CFSE labeled cells were then washed three times with PBS and cultured with or without stimulation.

### T cell activation

The 96-well assay plate precoated with anti-CD3 and anti-CD28 Ab (BD Pharmingen) was incubated at 37°C for 4 h. Splenocytes (1 ×10^6^ cells/well) were obtained from the spleens of naïve mice and were cultured for 3 days in 96-well plates in medium containing IL-2 (10 ng/mL; R&D Systems) as well as IL-12 (10 μg/mL; R&D Systems), or IL-4 (4 ng/mL; BD Pharmingen).

### Flow cytometric analysis

A single renal cell suspension was prepared and stimulated with PMA/Ionomycin/Golgi-plug for 4 h. The cells were incubated with different primary antibodies or the appropriate isotype control antibodies at 4°C for 30 min. The following antibodies were used PerCP/Cy5.5-conjugated anti-human CD14 (Biolegend), PerCP/Cy5.5-conjugated anti-mouse CD4 (Biolegend), APC-conjugated anti-mouse F4/80 (Biolegend), and PerCP/Cy5.5-conjugated anti-mouse CD11b (Biolegend). After cellular surface staining, cells were fixed and permeabilized with Cytofix/Cytoperm Soln Kit for intracellular staining with Alexa Fluor 488-conjugated anti-human C3 (Abcam) and PE-conjugated anti-mouse IL-17A (eBioscience). All flow cytometric analyses were performed using an LSR II Flow Cytometer (Beckman-Coulter) and Flowjo software.

### ELISA

To quantify IL-17A levels in the kidney, samples were analyzed using a mouse IL-17A ELISA (R and D Systems) according to the manufacturer's instructions. All measurements were performed in duplicate.

### Real-time PCR

Real-time PCR was performed as previously described (22). Real-time PCR was carried out using the LightCycler 480 system (Roche, Pleasanton, CA, USA) with the following primers: mouse C3, forward 5′-ACTGTGGACAACAACCTACTGC-3′, reverse 5′-GCATGTTCGTAAAAGGCTCGG-3′; mouse IL-6, forward 5′-TAGTCCTTCCTACCCCAATTTCC-3′, mouse reverse 5′-TTGGTCCTTAGCCACTCCTTC-3′; mouse IL-1β, forward 5′-GAAATGCCACCTTTTGACAGTG-3′, reverse5′-TGGATGCTCTCATCAGGACAG-3′; mouse TNF-α, forward 5′-CCTGTAGCCCACGTCGTA G-3′, reverse 5′-GGGAGTAGACAAGGTACAACCC-3′; mouse MCP-1, forward 5′-TTAAAAACCTGGATCGGAACCAA-3′, reverse 5′- GCATTAGCTTCAGATTTACGGGT-3′; mouse Collagen 1, forward 5′-GTCCTAGTCGATGGCTGCTC-3′, reverse 5′-CAATGTCCAGAGGTGCAATG-3′; mouse α-SMA, forward 5′-GGAGAAGCCCAGCCAGTCGC-3′, reverse 5′-AGCCGGCCTTACAGAGCCCA-3′; mouse PDGFR-β, forward 5′-GGGTCCGTTCCAGAAAATGT-3′, reverse 5′-GACAAGGGACCGGGGTCCAA-3′; mouse Arginase, forward 5′-AGCGCCAAGTCCAGAACCATA-3′, reverse 5′-CCATGCAAGTTTCCACTTGT-3′; mouse iNOS, forward 5′-TGGAGC GAGTTGTGGATTGTC-3′, reverse 5′-GTGAGGGCTTGGCTGAGTGA-3′; mouse GAPDH, forward 5′-AGGTCGGTGTGAACGGATTTG-3′, reverse 5′-GGGGTCGTTGATGGCAACA-3′; mouse C3, forward 5′-ACGGCATCCTCTGTCATCT-3′, reverse 5′-ACGGCATCCTCTGTCATCT-3′; TGF-β1, forward 5′-CGCAACAACGCCATCTATGA-3′, reverse 5′-ACCAAGGTAACGCCAGGAAT-3′. The relative amounts of mRNA were normalized to GAPDH and were calculated using the 2^−ΔΔ^Ct approach as previously reported (23).

### Statistical analysis

All statistical analyses were conducted using SPSS 12.0 (SPSS, USA). The values are expressed as the mean ± SEM. Graphpad Prism 5 software (GraphPad Software, La Jolla, CA, USA) was used for the statistical analysis with Student's *t*-test or one-way ANOVA where appropriate. The threshold for statistical significance was set at *P* < 0.05.

## Results

### Renal complement C3 expression is elevated and correlated with infiltrating CD68^+^ monocytes/macrophages in human IgAN biopsies

**Figure 1:**
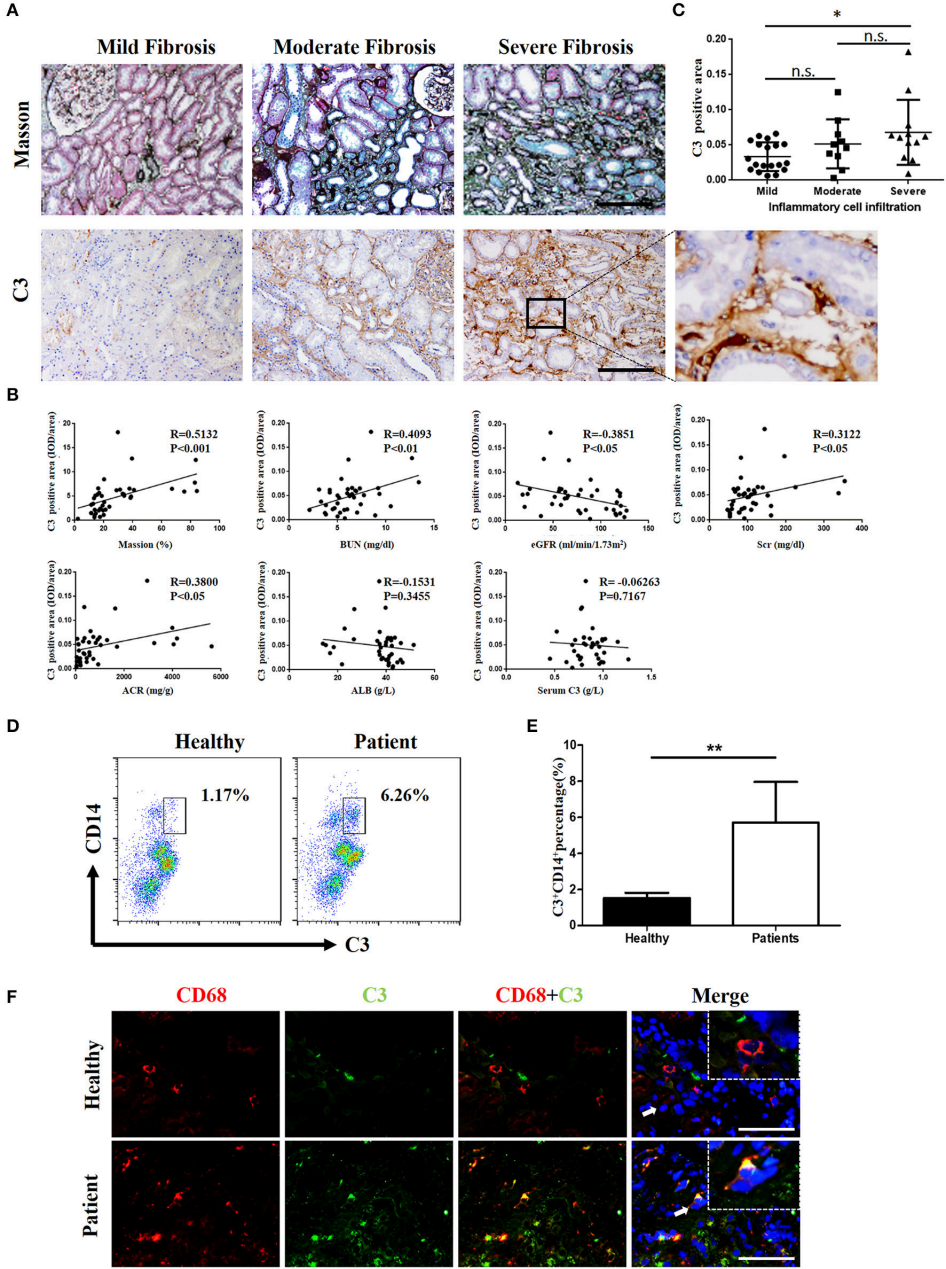
C3 levels were increased in renal tissues and blood samples from patients with IgAN **(A)** Masson's trichome staining showing collagen deposition; IHC analysis of C3 expression in the kidney. *n* = 41 patients with IgAN. **(B)** Relationships between C3 in the interstitium with Masson's trichome staining, BUN, the eGFR, Scr, ACR, ALB, and serum C3. **(C)** Relationships between C3 in the interstitium with the degree of inflammatory cell infiltration. Error bars represent SEM. ^*^P < 0.05. **(D)** Flow cytometric analysis showing the percentages of C3^+^CD14^+^ in PBMC of patients with IgAN. The data shown are representatives of FACS profiles. **(E)** The histograms show the increased the percentages of C3^+^CD14^+^ in peripheral blood mononuclear in the IgA patients (*n* = 41) compared with the healthy (*n* = 37). The error bars represent the SEM. ^**^P < 0.01. **(F)** Immunofluorescence staining of CD68 (red) and C3 (green) in the kidney. Scale bar, 50 μm.

Complement was previously shown to play a key role in IgAN pathogenesis, which involves the aberrant activation of the classic, alternative, and mannose-binding lectin pathways. We recruited IgAN patients whose renal biopsy specimens were reassessed blindly by a single pathologist using the Oxford classification. Notably, in renal biopsy specimens, C3 expression was observed in both renal tubules and the interstitium, and a positive correlation was found between pathologist-assessed Masson's trichrome staining and C3 expression, although the correlation between C3 expression in the interstitium and serum C3 was not statistically significant (Figures 1A,B). However, the intensities of C3 in the interstitium were significantly positively correlated to BUN, serum creatinine (SCr), and urine proteinuria/UCr (ACR) and negatively correlated to the eGFR (Figure 1B). No correlation was found between the intensities of C3 in the interstitium and ALB. In addition, along with exacerbated RF and enhanced mononuclear leukocyte infiltration, C3 expression increased significantly (Figure 1C).

However, evidence of local secretion of complement components by infiltrating cells during IgAN is still absent. To address this, we assessed C3 expression in renal tissues and performed flow cytometric analysis of C3 secretion in peripheral blood of patients with IgAN. C3 secretion was increased significantly in peripheral blood monocytes from IgAN patients (Figures 1D,E). Accordingly, C3 expression and monocyte infiltration were examined in paraffin-embedded sections of IgAN tissues by immunofluorescence staining. C3 expression was detected in the interstitium with significant co-staining of macrophages, which was observed as double-positive cells in patients, while healthy individuals showed little or no co-staining (Figure 1F). Altogether these results suggest that infiltrating macrophages as well as monocytes are a major source for C3 synthesis in kidney tissue.

### Renal expression of C3 is upregulated in the mouse UUO model

**Figure 2:**
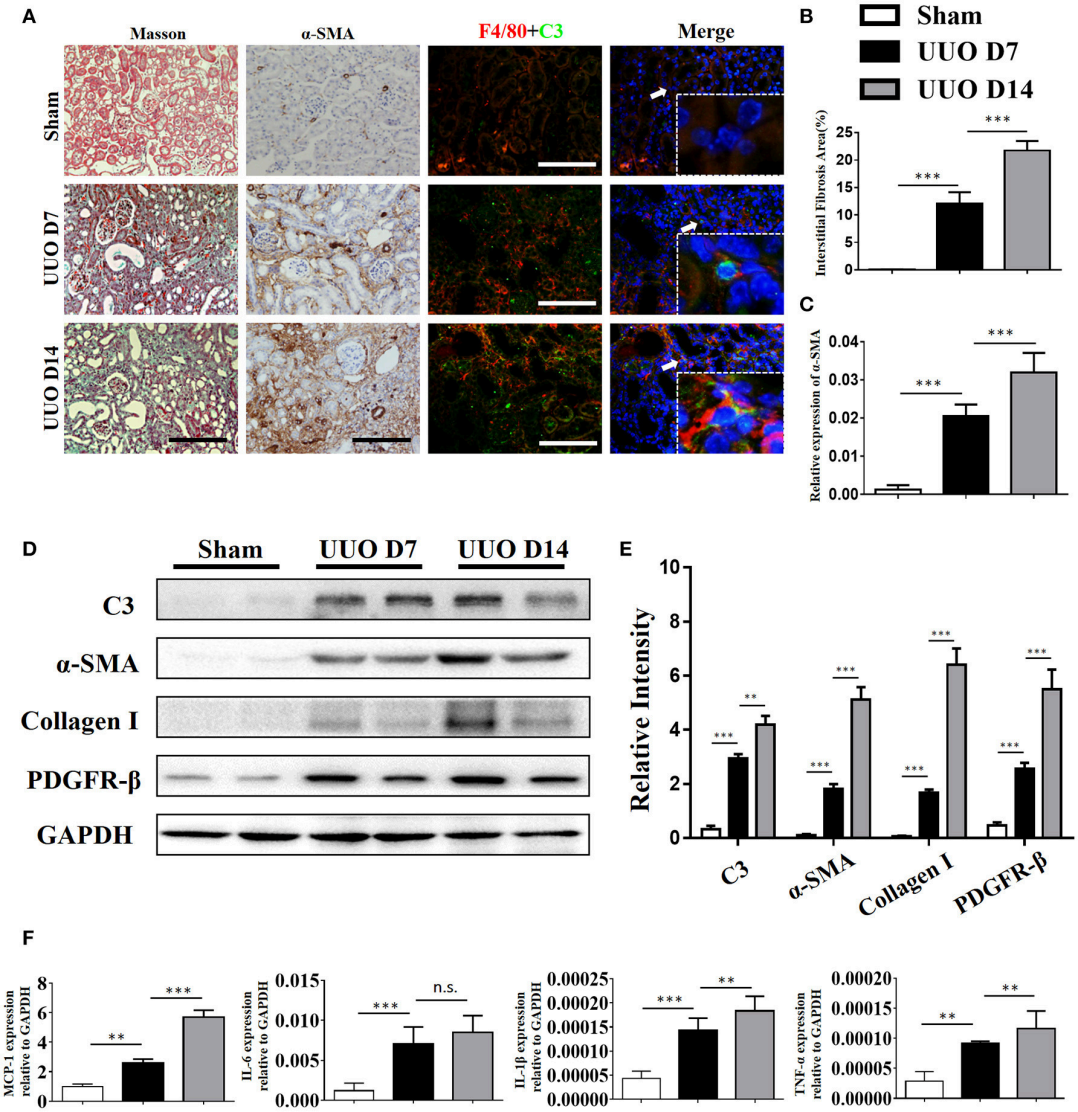
Complement C3 is increased in the obstructed kidney. **(A)** Masson's trichrome staining indicates collagen deposition. IHC staining showing α-SMA expression in the sham control and UUO mice; Immunofluorescence staining showing increased C3 (green) expression in UUO kidneys in the interstitium on days 7 and 14 and colocalization (white arrows) with F4/80 (red) original magnification, ×400. Quantitative positive area of interstitial fibrosis **(B)** and a-SMA **(C)** positive were shown as mean ± SEM. *n* = 6 per group, Scale bar, 50 μm. **(D)** The expression levels of C3 and fibrotic markers (α-SMA, PDGFR-β, and Collagen I) were detected by Western blot, and **(E)** the histogram shows the relative intensity for each marker normalized to GAPDH. *n* = 6 per group. **(F)** Real-time PCR showing relative renal mRNA levels of MCP-1, TNF-α, IL-6, and IL-1β in sham and UUO mice. The error bars represent the SEM. ^**^P < 0.01; ^***^P < 0.001.

C3 deposits within the glomerulus have been well-characterized in previous studies. To assess whether expression of C3 in the renal interstitium and kidney tubules is upregulated following UUO-induced renal injury, we performed immunochemical staining, real-time PCR, and Western blot analyses to measure its expression. Extensive stromal fibrosis was detected by Masson's trichrome staining, and increased C3 synthesis, α-SMA expression, and infiltrating macrophages were detected by immunohistochemistry (IHC) in mouse kidneys after the operation compared with those of the sham-operated mice; these parameters peaked on day 14 (Figures 2A–C, Supplement Figures 1B–E). Consistent with the histopathological results, C3 mRNA (Supplement Figure 1A) and protein (Figures 2D,E) levels were significantly increased after 7 and 14 days of UUO. Meanwhile, elevated MCP-1, IL-6, IL-1β, and TNF-α mRNA expression was also observed in UUO mice (Figure 2F).

### Macrophages are the major source of complement C3 production in the kidney following obstructive injury

**Figure 3:**
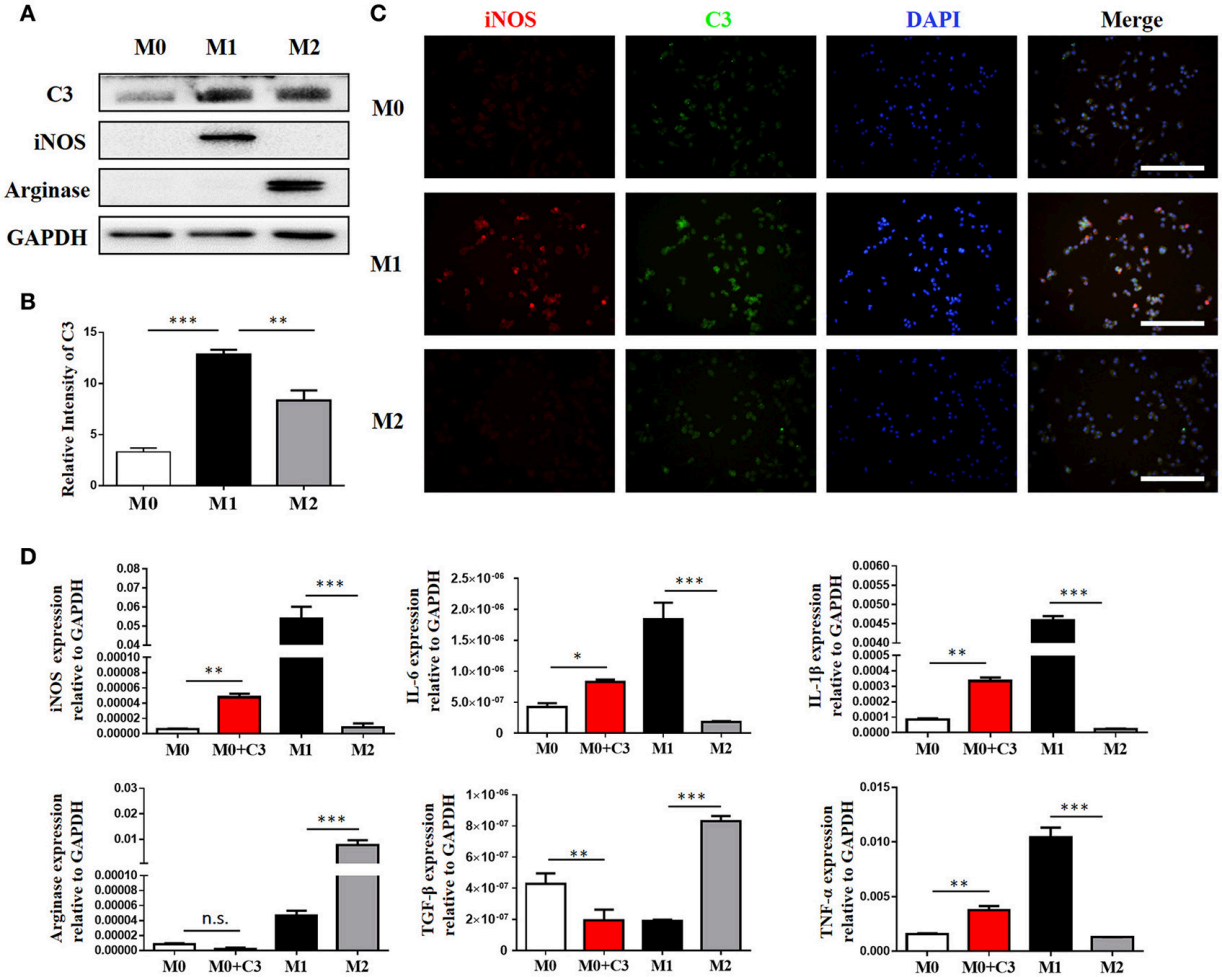
Macrophages are the major source of C3 in the obstructed kidney. **(A)** The protein isolated from BMMs treated for 24 h with either IFN-γ or IL-4 were analyzed by Western blot analyses, and **(B)** the histogram shows the relative intensity for each marker normalized to GAPDH. **(C)** Immunofluorescence staining showing increased C3 (green) primarily expressed in M1 with colocalization with iNOS (red) (original magnification ×400). Scale bar, 50 μm. **(D)** The effect of exogenous C3 on the subtype of macrophage by real-time PCR. The histogram shows the relative mRNA levels of iNOS, IL-6, IL-1β, Arginase, TGF-β, and TNF-α in these groups. *n* = 3. The error bars represent the SEM. ^*^P < 0.05; ^**^P < 0.01; ^***^P < 0.001. The data were pooled from three independent experiments.

Our IHC studies showed C3 overexpression in the interstitium of obstructed kidneys. To further define which cells were the major source of C3 production in UUO kidneys, we performed immunofluorescence staining using CD3, ly6G, F4/80, and α-SMA to identify T cells, PMNs, macrophages, and myofibroblasts, respectively. Figure 2A shows macrophages detected in the interstitium with significant C3 co-staining, shown as double-positive cells, in UUO mice, while the sham group showed little or no co-staining. Given that macrophages with different activation phenotypes play distinct roles, we then proceeded to verify which subset of macrophages expressed C3 *in vitro*. Bone marrow-derived macrophages (BMDMs) were assessed after 7 days of culture with L929 supernatant. As previously reported, classically activated macrophages (M1) expressed high levels of iNOS and little Arginase-I, and alternatively activated macrophages (M2) expressed high levels of Arginase-I and little iNOS (Figure 3A). We found that C3 was primarily expressed by M1 cells, as shown by immunofluorescence staining and Western blotting (Figures 3B,C). Our results show that both tubules and interstitial cells secrete C3. Macrophages in the interstitium are likely to be affected in the microenvironment. As shown in Figure 3D, the addition of C3 to the induced differentiated macrophages *in vitro* could stimulate increased iNOS, IL-6, and IL-1β expression and decreased Arginase-I, TGF-β, and TNF-α expression, indicating that C3 could induce macrophage differentiation into M1.

### Macrophage depletion reduces complement expression and renal fibrosis

**Figure 4:**
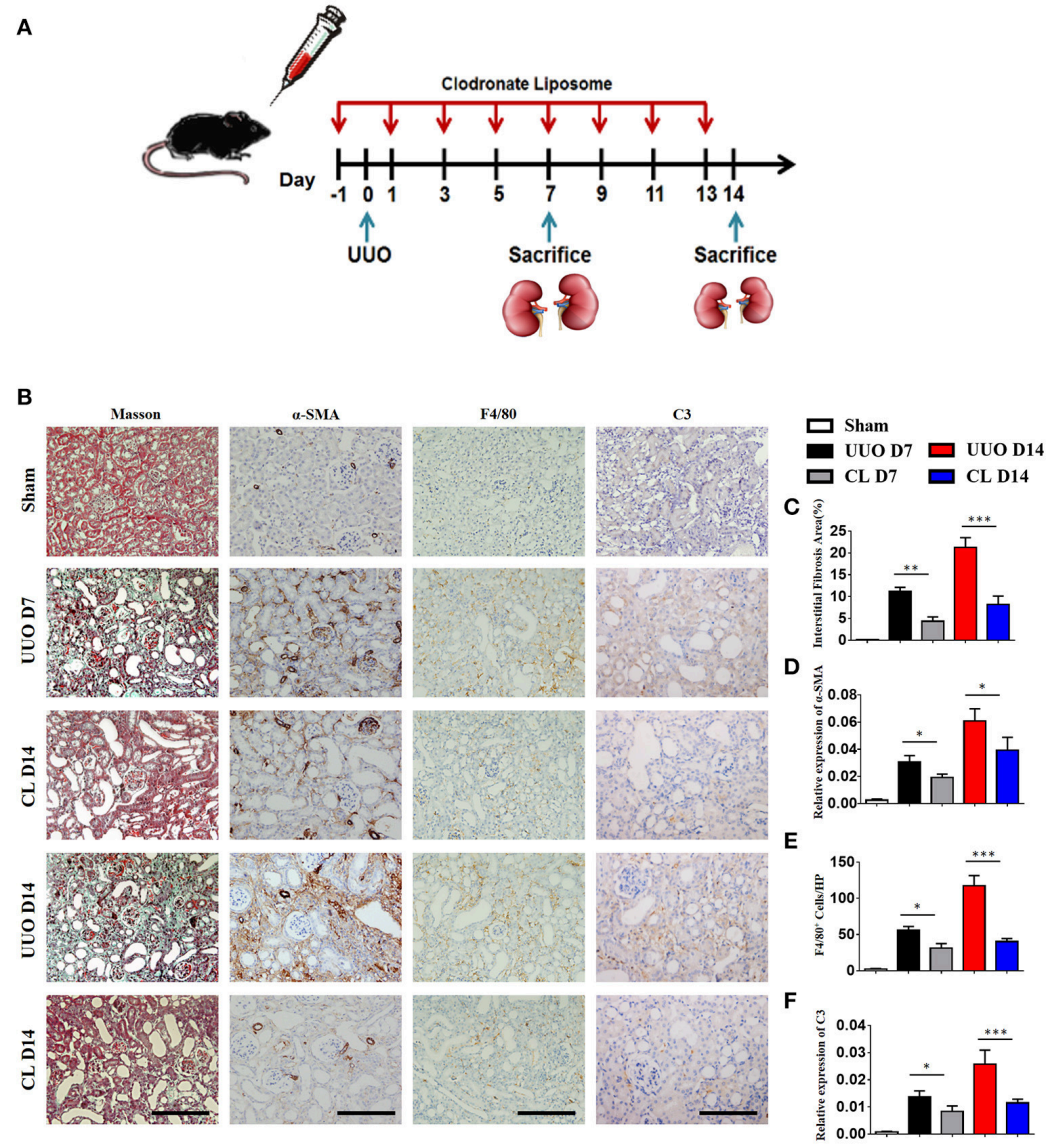
Macrophage depletion protects against renal fibrosis by inhibiting C3 expression. UUO mice were intravenously injected with clodronate liposomes and control liposomes. On days 7 and 14, mice were sacrificed, and the left kidneys were collected. **(A)** Scheme of clodronate liposome-mediated macrophage depletion. **(B)** Masson's trichrome staining indicates collagen deposition. IHC staining showing α-SMA, F4/80, and C3 protein expression in these groups (*n* = 6); original magnification, ×400. Scale bar, 50 μm. Quantitative analysis of interstitial fibrosis**(C)**, a-SMA**(D)**, F4/80 **(E)**, and C3 **(F)** positive cells were shown as mean ± SEM. ^*^P < 0.05; ^**^P < 0.01; ^***^P < 0.001.

Our above results showed that macrophages, especially M1 macrophages, contribute significantly to C3 secretion. We depleted F4/80^+^ macrophages using clodronate liposomes in UUO mice. As depicted in Figure 4A, mice were sacrificed after 7 and 14 days of UUO, and the results showed that macrophages were reduced in UUO mice injected with clodronate compared to those injected with control PBS liposomes. Similarly, UUO mice treated with clodronate exhibited significantly decreased C3 expression. The reduction in C3 expression was associated with reduced α-SMA expression and decreased tubulointerstitial fibrosis measured by Masson's trichrome staining (Figures 4B–F). In addition, the mRNA and protein levels of C3, α-SMA, and PDGFR-β were reduced in UUO mice receiving clodronate liposomes (Supplement Figure 2). These results further support the pathogenic function of macrophage infiltration with increased C3 expression, which leads to RF.

### C3 deficiency attenuates fibrosis and infiltration of inflammatory cells in UUO-induced renal fibrosis

**Figure 5:**
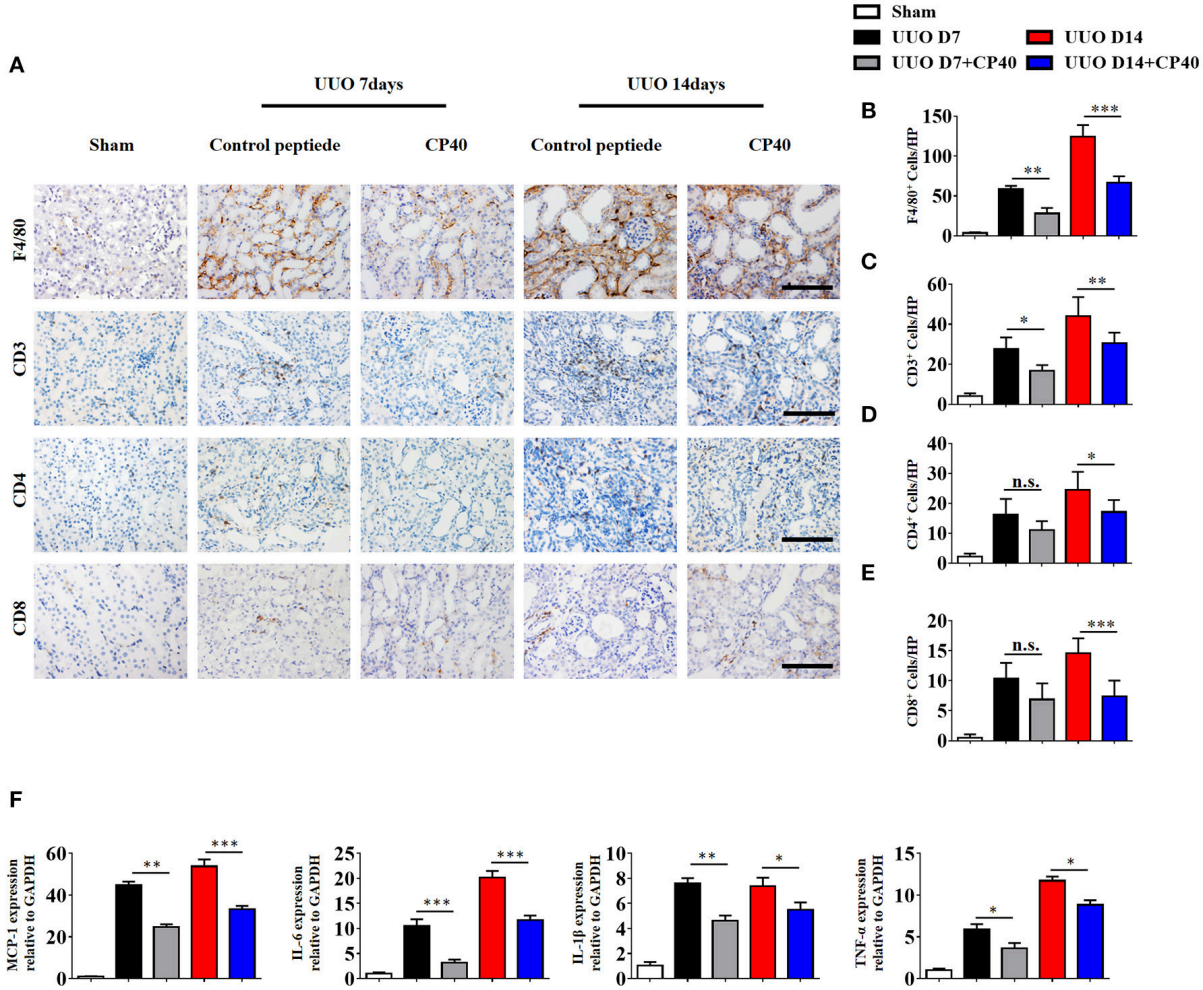
C3 deficiency reduces C3 expression, inflammatory infiltration and fibrosis in kidney during UUO. UUO mice were subcutaneously injected with control peptide and Cp40. On days 7 and 14, mice were sacrificed, and the left kidneys were collected. **(A)** IHC staining showing F4/80 macrophages, CD3^+^T cells, CD4^+^T cells, and CD8^+^T cells in these groups (*n* = 6); original magnification, ×400. Scale bar, 50 μm. Quantitative analysis of F4/80 **(B)**, CD3 **(C)**, CD4 **(D)**, and CD8 **(E)** positive cells were shown as mean ± SEM. **(F)** Real-time PCR showing relative renal levels of MCP-1, IL-6, IL-1β, and TNF-α in these groups (*n* = 6). The error bars represent the SEM. ^*^P < 0.05; ^**^P < 0.01; ^***^P < 0.001.

To investigate the role of C3 in the pathogenesis of UUO, we used a peptidic C3 inhibitor, Compstatin analog Cp40, to block C3 activation. Masson's staining and α-SMA expression analysis showed that UUO mice injected with 1 mg/kg Cp40 had much less severe interstitial fibrosis than control peptide-injected mice (Supplement Figures 3A–D). Western blot analysis also indicated that the α-SMA and PDGFR-β levels were decreased in the Cp40-injected UUO mice (Supplement Figures 3E,F). In addition to the attenuated tubulointerstitial fibrosis, renal infiltration of F4/80^+^ macrophages, CD3^+^T cells, CD4^+^T cells, and CD8^+^T cells was significantly reduced in Cp40-injected UUO mice compared with peptide-injected mice (Figures 5A–E). Meanwhile, elevated MCP-1, IL-6, IL-1β, and TNF-α mRNA expression in UUO mice was markedly limited by Cp40 (Figure 5F). These data indicate that C3 mediates the infiltration of T cells and macrophages into the kidney in response to obstructive injury.

### C3aR blockade substantially attenuates renal fibrosis in UUO mice

**Figure 6:**
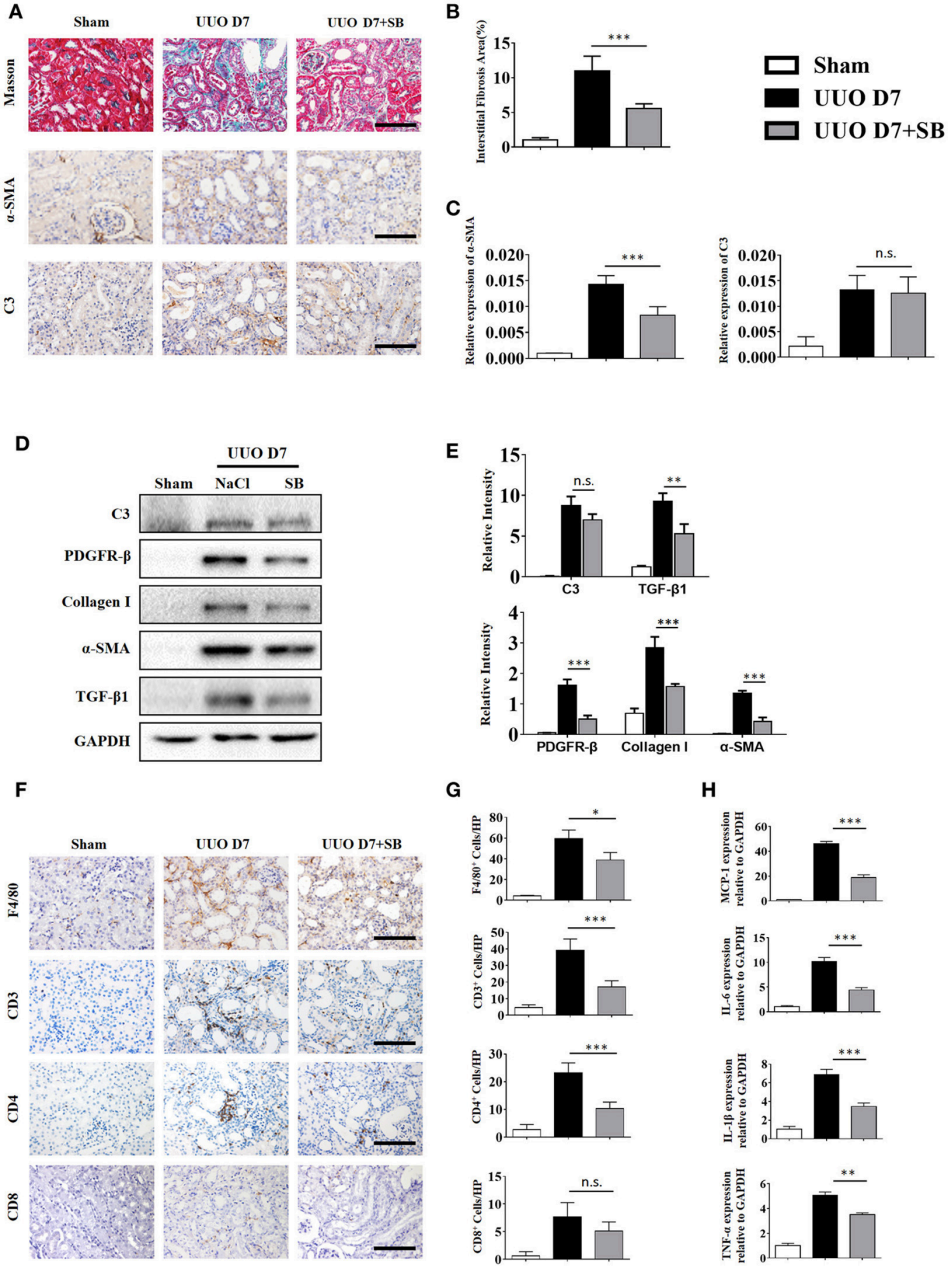
C3aR blockade reduces inflammatory infiltration and fibrosis during UUO. UUO mice were intraperitoneally injected with vehicle and SB290157. On day 7, mice were sacrificed, and the left kidneys were collected. **(A)** Masson's trichrome staining indicates collagen deposition. IHC staining showing α-SMA and C3 protein expression in these groups (*n* = 6); original magnification, ×400. Scale bar, 50 μm. Quantitative analysis of interstitial fibrosis **(B)**, a-SMA **(C)**, and C3 positive cells **(C)** were shown as mean ± SEM. **(D)** The expression levels of C3, TGF-β1, and fibrotic markers (α-SMA, PDGFR-β, and Collagen I) were detected by Western blot. **(E)** The histogram shows the relative intensity for each marker normalized to GAPDH. **(F)** IHC staining showing F4/80 macrophages, CD3^+^T cells, CD4^+^T cells, and CD8^+^T cells in these groups (*n* = 6); original magnification, ×400. Scale bar, 50 μm. **(G)** Quantitative analysis of F4/80, CD3, CD4, and CD8 positive cells were shown as mean ± SEM. **(H)** Real-time PCR showing relative renal levels of MCP-1, IL-6, IL-1β, and TNF-α in these groups (*n* = 6). The error bars represent the SEM. ^*^P < 0.05; ^**^P < 0.01; ^***^P < 0.001.

C3a is one of the proteins formed by the cleavage of C3 and plays a large role in the immune response. Given that increased generation of C3 was observed in IgAN patients and UUO mice, we proceeded to investigate whether the renal parenchymal loss in UUO mice could be attenuated by C3aR antagonism, SB290157. SB290157 is a selective antagonist of complement anaphylatoxin C3a receptor, a 74 amino acid proinflammatory mediator and chemotactic peptide. It effectively blocks C3aR in humans, rat, guinea pig, and mouse. As shown in Figures 6A–C, the progression of renal interstitial fibrosis was dramatically retarded by daily i.p., injection of SB290157, which was initiated on day 7 after the UUO operation. And injection of SB290157 did not affect C3 expression in renal interstitium of UUO mice. Similarly, along with attenuated RF and TGF-β1 release were significantly reduced by treatment with C3aRA (Figures 6D,E>). In addition, we showed that renal infiltration of F4/80^+^macrophages, CD3^+^T cells, CD4^+^T cells, and CD8^+^T cells was significantly reduced in SB290157-injected UUO mice compared with that in peptide-injected mice (Figures 6F–G). Additionally, elevated MCP-1, IL-6, IL-1β, and TNF-α mRNA expression in UUO mice was markedly limited by SB290157 (Figure 6H). Taken together, these data suggest C3-C3aR signaling promotes RF, and C3aR may be a therapeutic target for renal disease.

### Blocking C3-C3aR signaling attenuates renal fibrosis by inhibiting IL-17A production in UUO mice

**Figure 7:**
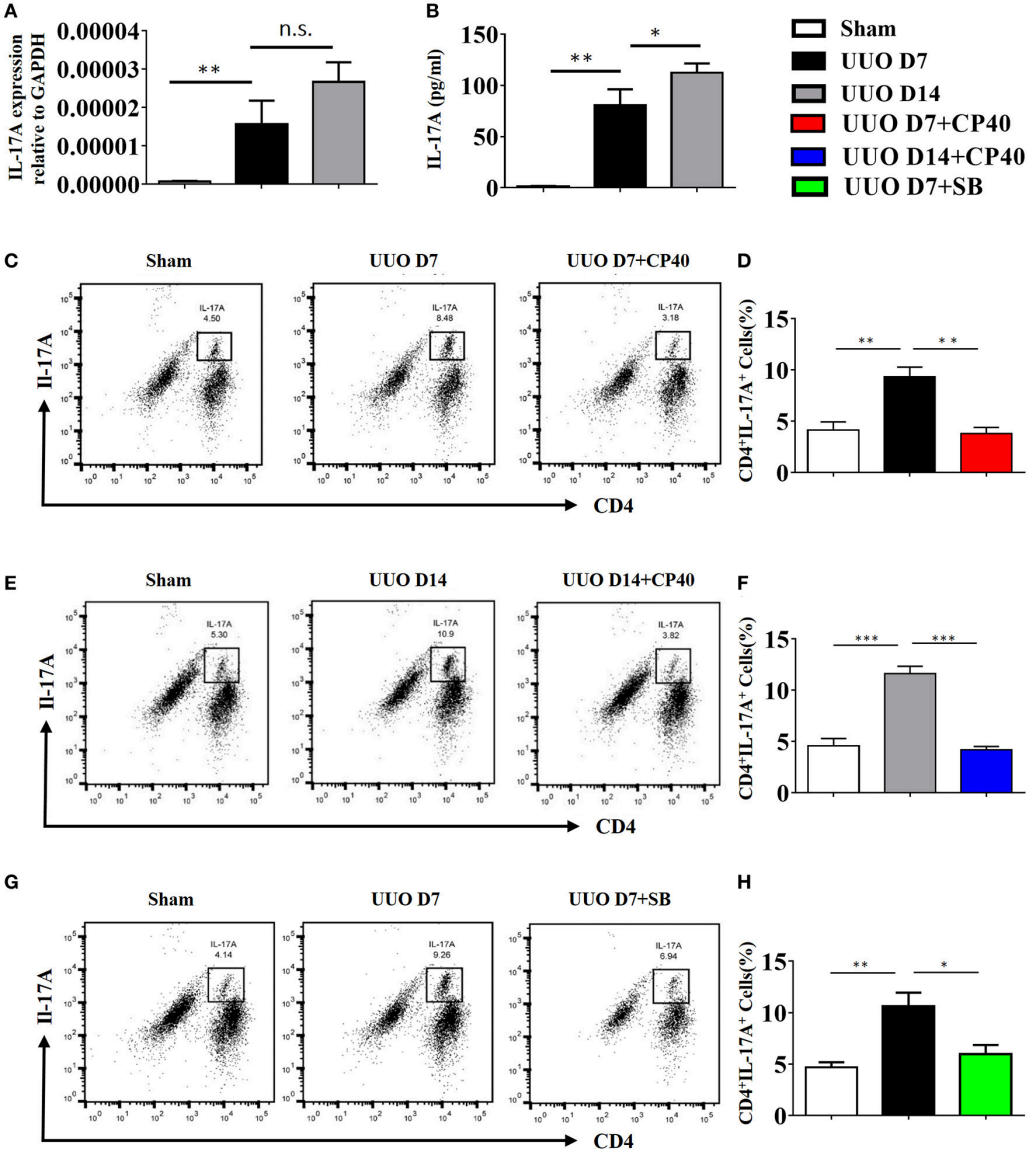
The expression of IL-17A is induced in UUO mice. Mice were subjected to unilateral ureteral obstruction (UUO) by left ureteral ligation for 7 and 14 days (*n* = 6 per group). **(A)** IL-17A mRNA in whole kidneys measured by real-time PCR; ^**^P < 0.01 vs. sham operation; *n* = 6. **(B)** Production of IL-17A in obstructed kidneys was determined by CBA and was normalized to total protein content for each sample; ^**^P < 0.01, ^*^P < 0.05 vs. sham operation; *n* = 6. Flow cytometric analysis **(C,E,G)** and quantification **(D,F,H)** of kidney cell suspensions from the obstructed kidneys injected with or without Cp40/SB290157 during the time course of UUO; *n* = 6. Cells were stimulated *in vitro* with PMA/Ionomycin/Golgi-plug for 4 h. Specific staining of cell markers (anti-CD4) and intracellular staining for IL-17A were performed. Plots are gated for live CD4^+^IL-17A^+^lymphocytes; numbers indicate events in the quadrants as percentages of all gated events. The error bars represent the SEM. ^*^P < 0.05; ^**^P < 0.01; ^***^P < 0.001. The data were pooled from three independent experiments.

As shown in Figure 7A, renal mRNA levels of IL-17A were substantially increased in UUO mice compared with sham control mice. In addition, IL-17A levels in the serum of UUO mice were significantly increased in the early and late stages compared with those in the serum of sham control mice (Figure 7B). Consistent with the ELISA and mRNA data, the FACS results revealed that 8.48 and 10.9% of CD4^+^ renal cells in obstructed kidneys expressed IL-17A following UUO, respectively, whereas ≤5.3% were IL-17A^+^ in sham-operated mice, and this effect was strongly inhibited by Cp40 and SB290157 (Figures 7C–H). In addition, we performed analysis of CD11b^+^F4/80^+^IL-17^+^ cells ratio in kidney from C3 blockade UUO mice and UUO mice. The results showed that CD11b^+^F4/80^+^IL-17^+^cells were around 1% in mononuclear cells in two groups, and only slightly changed after blockade C3 with CP40 (Supplement Figures 4A,B). Similar results were confirmed in 14 days of UUO mice (Supplement Figures 4C,D). Thus, we identified that the main producer of IL-17A in the UUO mice were T cells, which were strikingly increased after unilateral ureteral ligation.

**Figure 8:**
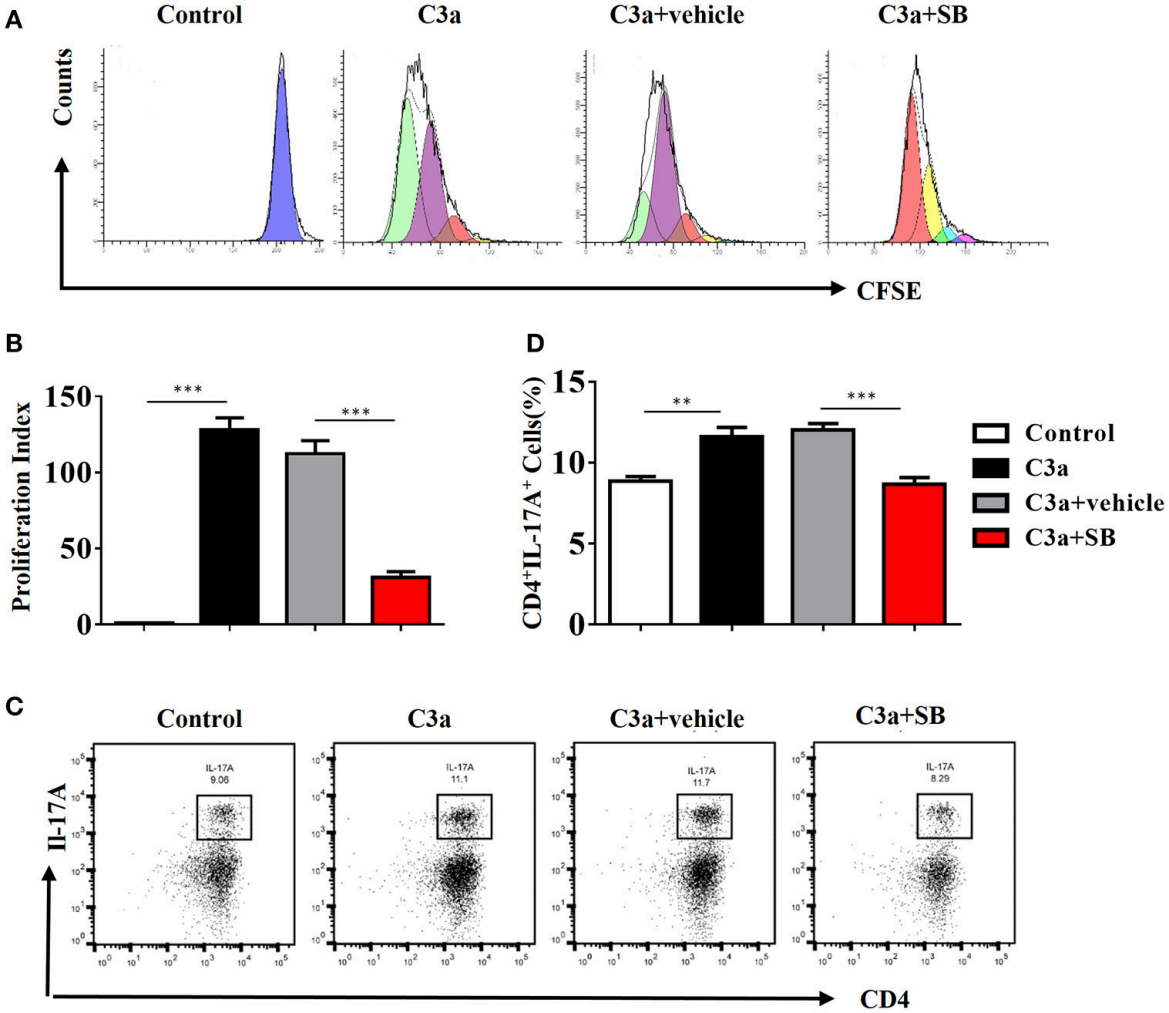
C3a acts as regulator for renal IL-17A. Splenocytes stimulated with recombinant C3a (20 nM) for 72 h in the presence or absence of SB290157. **(A)** Average T cell proliferation increased in response to mC3a, while T cell proliferation decreased in SB290157-treated cultures compared to those containing only mC3a. **(B)**The histograms show the blockade of C3a drastically suppressed T cell proliferation stimulated with C3a. The error bars represent the SEM. ^***^P < 0.001. **(C)** Splenocytes were double-stained for CD4 and IL-17A. Representative dot plots are presented, and the numbers in the quadrants represent the percentage of each population. **(D)** Quantifications of CD4^+^IL-17A^+^cells as percentages of splenocytes isolated from the WT mice. The error bars represent the SEM. ^**^P < 0.01; ^***^P < 0.001. The data were pooled from three independent experiments.

To investigate the mechanism by which C3a promotes IL-17A expression, we isolated spleen cells from naïve wild-type mice on anti-CD3/anti-CD28-coated plates *in vitro*. We extended our CFSE dilution assay using splenocytes, and T cells exhibited high levels of proliferation in the presence of mC3a. Inhibition by SB290157 abrogated T cell proliferation; therefore, mC3a is required for T cell proliferation (Figures 8A,B). More importantly, blockade of C3a by the C3aR inhibitor drastically suppressed IL-17A expression in C3a-stimulated T cells (Figures 8C,D). In this study, we found that C3 deficiency significantly reduced the IL-17A production in obstructed kidneys. Given that IL-17A can stimulate chemokine expression in renal and immune cells, our findings establish C3a as a mediator by which IL-17A initiates infiltration of inflammatory cells during obstructive injury. Thus, complement C3 activation represents a key event for triggering the production of IL-17A during obstructive injury, thereby shaping renal microenvironments.

**Figure 9:**
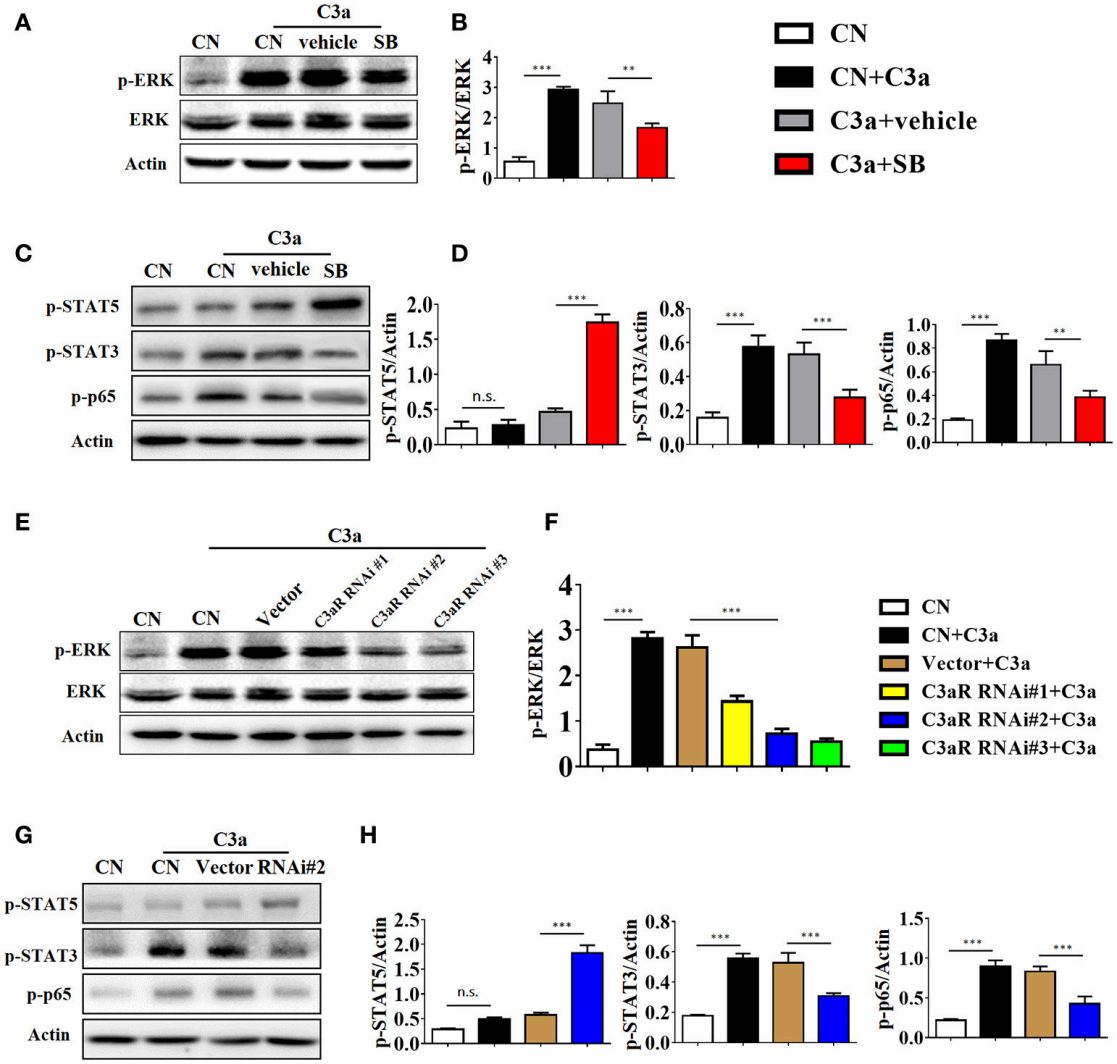
C3a/C3aR signaling promotes the IL-17A production in T cells *in vitro*. **(A,C)** Splenocytes stimulated with C3a (20 nM) for 30 min in the presence or absence of SB290157. Extracellular signal-regulated kinase-1/2 (ERK1/2), signal transducers and activators of transcription-3/5 (STAT3/5), and nuclear factor (NF)-kB p65 subunit phosphorylation were detected by Western blotting. **(B,D)** The histogram shows the relative intensity for each marker normalized to Actin. **(E,G)** T cells were knocked down endogenous C3aR by using siRNAs, and stimulated with C3a (20 nM) for 30 min, p-ERK, p-STAT3, p-STAT5, and p-p65 were detected by Western blotting. **(F,H)** The histogram shows the relative intensity for each marker normalized to Actin. The error bars represent the SEM. ^**^P < 0.01; ^***^P < 0.001.

As the ERK signaling pathway was reported to be indispensable for C3a-mediated effector responses during kidney transplant, we defined the role of ERK signaling in IL-17A release upon C3a stimulation. The results showed that exposure to C3a led to phosphorylation of ERK, STAT3, and STAT5 and activation of NF-kB in T cells (Figures 9A–D). To further conformed the impact of C3a on activation of ERK signaling pathway, we knocked down endogenous C3aR in T cells by using specific small-interfering RNAs (siRNAs). As shown in Supplement Figure 5, two siRNAs targeting C3aR specifically knocked down endogenous C3aR protein in T cells. siRNA # 2 with higher efficiency was chosen for the subsequent studies. The results showed that T cells depleted in C3aR can inhibit phosphorylation of ERK, STAT3, and STAT5 and activation of NF-kB, which active by C3a (Figures 9E–H). Taken together, our data indicated that C3a could induce ERK and STAT3 signaling pathway activation and promote the IL-17A production in T cells *in vitro*.

**Figure 10:**
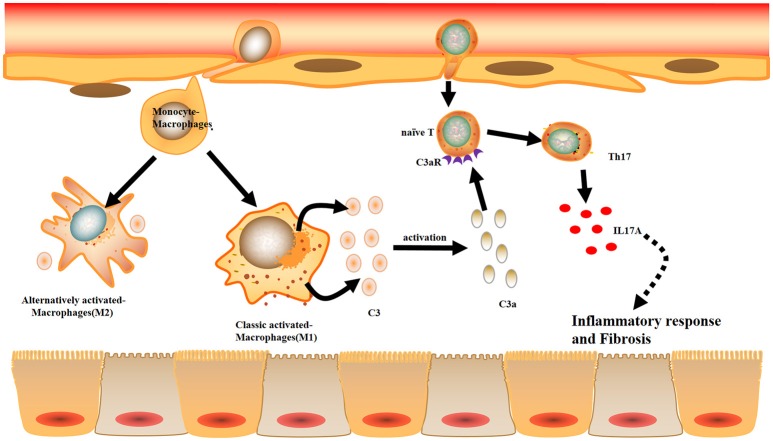
Model summarizing the role of local C3 in the development of renal fibrosis. Local C3 secretion by macrophages may be contributed to the pathogenesis of renal TILs, involving the production of IL-17A and activation of ERK and STAT3 signaling pathway.

Combined with the data obtained from the mouse and cell experiments, the results obtained from IgAN patients further support the notion that local C3 secretion by macrophages leads to renal fibrogenic responses (Figure 10).

## Discussion

In human samples, we showed that circulating and local C3 was highly expressed in peripheral blood and renal tissues from patients with IgAN. Then, we observed that most of the C3 deposited in the interstitium and its expression were associated with the severity of renal interstitial fibrosis. Previous studies have predominantly investigated C3 glomerulopathy, which is defined as a kidney disease caused by complement dysregulation that results in variable glomerular inflammation (24–26). In most of the cases, C3 deposition in the glomeruli, as shown by immunofluorescence, had no immunoglobulins due to alternative pathway activation. However, further elucidation of C3 deposition in the renal interstitium is needed for a better understanding of its initiation and exacerbation. In our study, the contribution of C3 secretion by macrophages that infiltrated in the kidney appeared to be more important than C3 in the intravascular space as shown by IHC and immunofluorescence in renal tissues. (1) Immunofluorescence of human patients showed that C3 expression was more intense in macrophages than parenchymal cells, and (2) C3 was barely expressed in renal tubules of UUO mice. We further showed that local C3 levels were highly correlated with RF. Consistent with these results, cultured mouse BMDMs under stimulations mimicking the inflammatory microenvironment, M1 and M2 all showed strongly increased secretion of C3. Furthermore, we found that C3 facilitates IL-17A production in T cells, contributing to the development of renal inflammation and fibrosis in UUO mice. In addition, blockage of C3 by Cp40 attenuated RF of UUO mice. Our proposed mechanistic network for the pathogenic role of C3 in the development of RF is also summarized in Figure 10.

Renal fibrosis is considered to be a common end point of various types of CKD, and its biological significance depends on the cell types contributing to collagenous and non-collagenous extracellular matrix production (27–33). The associated processes include vascular leakage, leukocyte recruitment, angiogenesis, and the appearance of myofibroblasts. Currently, most data have focused on the precursor cells of renal myofibroblasts, including circulating bone marrow-derived cells, or the transition from epithelial or endothelial cells, pericytes, and resident fibroblasts (34–39). However, these types of transdifferentiation were all triggered by innate and adaptive immune responses through production of proinflammatory and profibrotic molecules. As a model of tubulointerstitial fibrosis, UUO model was thought best for this approach, as it is a rapid and reproducible model of RF in mice, and it mimics the main steps of tubulointerstitial fibrosis in humans (40–42). In this study, we explored the interactions between the innate and adaptive immune systems in the process of promoting RF. In the early stage during the pathogenesis of RF, macrophages are present at the affected areas (43). As an essential component of innate immunity, macrophages also regulate adaptive immune responses by recruiting other immune cells, such as neutrophils, mast cells and lymphocytes (44–49). Our results confirmed that depletion of kidney macrophages by clodronate significantly attenuated RF and function in UUO models, which has been demonstrated by previous reports.

Macrophages are highly heterogeneous cells subdivided according to their distinct functions (50, 51). In CKD, M1 macrophages are increased during early injury and inflammation and persistently surround regions of damaged tissue. Subsequently, macrophages switch to an anti-inflammatory (M2) phenotype and contribute to resolution of inflammation (43). Our evidence showed that resident kidney macrophages can secrete C3, and M1 macrophages secrete higher amounts of C3 than M2 macrophages.

Prominent roles of CD4^+^T cells in chronic diseases have been reported in previous studies (52, 53). Our colleagues showed that massive CD4^+^T lymphocyte infiltration was observed in the fibrotic kidneys of patients and UUO mice (54). After antibodies were used to deplete CD4^+^ T cells, HE and Masson's trichrome staining results showed less inflammatory infiltrates and attenuated interstitial fibrosis in CD4^+^T lymphocyte-depleted mice compared with UUO mice. When we depleted C3 and C3a, the infiltrating inflammatory cells, including macrophages, CD3^+^T cells, and CD4^+^T cells, decreased in UUO mice, as demonstrated by IHC and flow cytometric analyses, indicating that CD4^+^T cell differentiation occurs after UUO. The data suggest that the C3a/C3aR signaling pathway.

In inflammatory kidney diseases, IL-17A producing by T lymphocytes contributes significantly to the pathogenesis of RF (55). A newly-published study has shown that IL-17 acts an inhibitory factor in TGF-β-induced renal fibroblast activation using the UUO model with IL-17^−/−^mice (56). The explanation for this discrepancy might be that the IL-17 cytokine family consists of six members (IL-17A–F), and the potential effect of the other IL-17 family members in renal autoimmunity and inflammation is unknown. Another study has shown that macrophages and neutrophils are major source of IL-17 in different diseases (57–60). But Tamassia noted that the human neutrophils are unable to express and produce IL-17A, IL-17B, or IL-17F *in vitro* (61). Our data show that IL-17A was mainly produced by CD4^+^Tcell, rather than F4/80^+^macrophages in UUO mice, which was consistent with previous reports (55, 62).

In addition, IL-17A, which is secreted by CD4^+^T cells, has been shown to play a major role in post-transplantation allograft rejection and in immune responses in the kidney (63–65). In the presence of TGF-β and IL-6, T cells differentiate into Th17 cells. In this regard, we observed the expression of chemokines (MCP-1, IL-1β, and IL-6) and lymphocyte infiltration in the obstructed kidneys of mice on days 7 and 14 after UUO and IL-17A secretion by CD4^+^T cells in the UUO mice. The production of IL-17A is negatively regulated by the anaphylatoxins, and C3a signaling elevates Th17 responses, while C5a signaling suppresses Th17 cell differentiation in experimental allergic asthma. As illustrated in Figure 7, we found that C3a could promote proliferation of T cells and increase IL-17A secretion by CD4^+^T cell and activation of the ERK and STAT3 signaling pathways *in vitro*. These phenomena can be suppressed by a C3aR inhibitor, suggesting that the C3a/C3aR pathway participates in the pathogenesis of UUO injury by modulating IL-17A expression, consequently promoting local inflammation and RF.

Combined with the data obtained from patient samples, relevant animal experiments and cell models, our study defines a novel mechanism by which C3 participates in renal inflammation and fibrosis. In response to injury, local C3 secretion by macrophages leads to IL-17A-mediated inflammatory cell infiltration into the kidney, which further drives fibrogenic responses. Our findings suggest that inhibition of the C3a/C3aR pathway could constitute a novel therapeutic approach for obstructive nephropathy.

## Ethics statement

The study was approved by the Ethical Committee of Tongji Hospital, Tongji Medical College, Huazhong University of Science and Technology Institutional (Certificate Number: IRB ID:TJ-A20160201).

## Author contributions

The experiments were conceived and designed by YyL, XL, and CZ. Experiments were performed by KW, YL, YZ, and HW and data analyzed by KW, RL, and SG. The paper was written by YyL and GX with input from all authors.

### Conflict of interest statement

The authors declare that the research was conducted in the absence of any commercial or financial relationships that could be construed as a potential conflict of interest.

## Supplementary material

Supplement Figure S1 Complement C3 is increased in the obstructed kidney. **(A)** Real-time PCR showing relative renal mRNA levels of C3, fibrotic markers (α-SMA, PDGFR-β, and Collagen I) in sham control and UUO mice. **(B)** IHC staining showing F4/80 and C3 protein expression in the sham control and UUO mice; original magnification, ×400. Quantitative analysis of F4/80 **(C)** and C3 **(D)** positive cells were shown as mean ± SEM. *n* = 6 per group, Scale bar, 50 μm. **(E)** High magnification of C3 expression in IgA Patient and UUO mouse. The error bars represent the SEM. ^*^*P* < 0.05; ^**^*P* < 0.01; ^***^*P* < 0.001.

Supplement Figure S2 Macrophage depletion protects against renal fibrosis by inhibiting C3 expression. UUO mice were intravenously injected with clodronate liposomes and control liposomes. On days 7 and 14, mice were sacrificed, and the left kidneys were collected. The expression levels of C3 and fibrotic markers (α-SMA, PDGFR-β, and Collagen I) were detected by real-time PCR **(A)** and Western blot analyses **(B)**, respectively. **(C)**The histogram shows the relative intensity for each marker normalized to GAPDH. *n* = 6 per group. The error bars represent the SEM. ^*^*P* < 0.05; ^**^*P* < 0.01; ^***^*P* < 0.001.

Supplement Figure S3 C3 deficiency reduces C3 expression and fibrosis during UUO. UUO mice were subcutaneously injected with control peptide and Cp40. On days 7 and 14, mice were sacrificed, and the left kidneys were collected. **(A)** Masson's trichrome staining indicates collagen deposition. IHC staining showing α-SMA and C3 protein expression in these groups (*n* = 6); original magnification, ×400. Scale bar, 50 μm. Quantitative analysis of interstitial fibrosis **(B)**, a-SMA**(C)** and C3**(D)** positive cells were shown as mean ± SEM. **(E)** The expression levels of C3, TGF-β1, and fibrotic markers (α-SMA, PDGFR-β, and Collagen I) were detected by Western blot. **(F)** The histogram shows the relative intensity for each marker normalized to GAPDH. *n* = 6 per group. The error bars represent the SEM. ^*^*P* < 0.05; ^**^*P* < 0.01; ^***^*P* < 0.001.

Supplement Figure S4 CD11b^+^F4/80^+^macrophages are not the main producer of IL-17A in kidney of UUO mice. Flow cytometric analysis of kidney cell suspensions from the obstructed kidneys injected with or without Cp40 at **(A)** 7 days and **(C)** 14 days post UUO; *n* = 6. Cells were stimulated *in vitro* with PMA/Ionomycin/Golgi-plug for 4 h. Specific staining of cell markers (anti-F4/80, anti-CD11b) and intracellular staining for IL-17A were performed. The F4/80^+^ cells were gated. Among them, the CD11b^+^IL-17^+^cells were further gated for the analysis. Plots are gated for live CD11b^+^F4/80^+^ IL-17A^+^ macrophages; numbers indicate events in the quadrants as percentages of all gated events. **(B,D)** Quantifications of CD11b^+^F4/80^+^ IL-17A^+^ cells as percentages of all kidney cells isolated from the C3 blockade UUO mice and UUO mice. The error bars represent the SEM. ^***^*P* < 0.001. The data were pooled from three independent experiments.

Supplement Figure S5 T cells were knocked down endogenous C3aR by using three small-interfering RNAs (siRNAs). **(A)** The expression levels of C3aR was detected by Western blot. **(B)** The histogram shows the relative intensity for each marker normalized to Actin. *n* = 3 per group. The error bars represent the SEM. ^***^*P* < 0.001.
