# Supplementary figures and images for: Complement C3 Produced by Macrophages Promotes Renal Fibrosis via IL-17A Secretion
Source: Front Immunol. 2018 Oct 22;9:2385. doi: 10.3389/fimmu.2018.02385 (PMC6204358; doi:10.3389/fimmu.2018.02385)

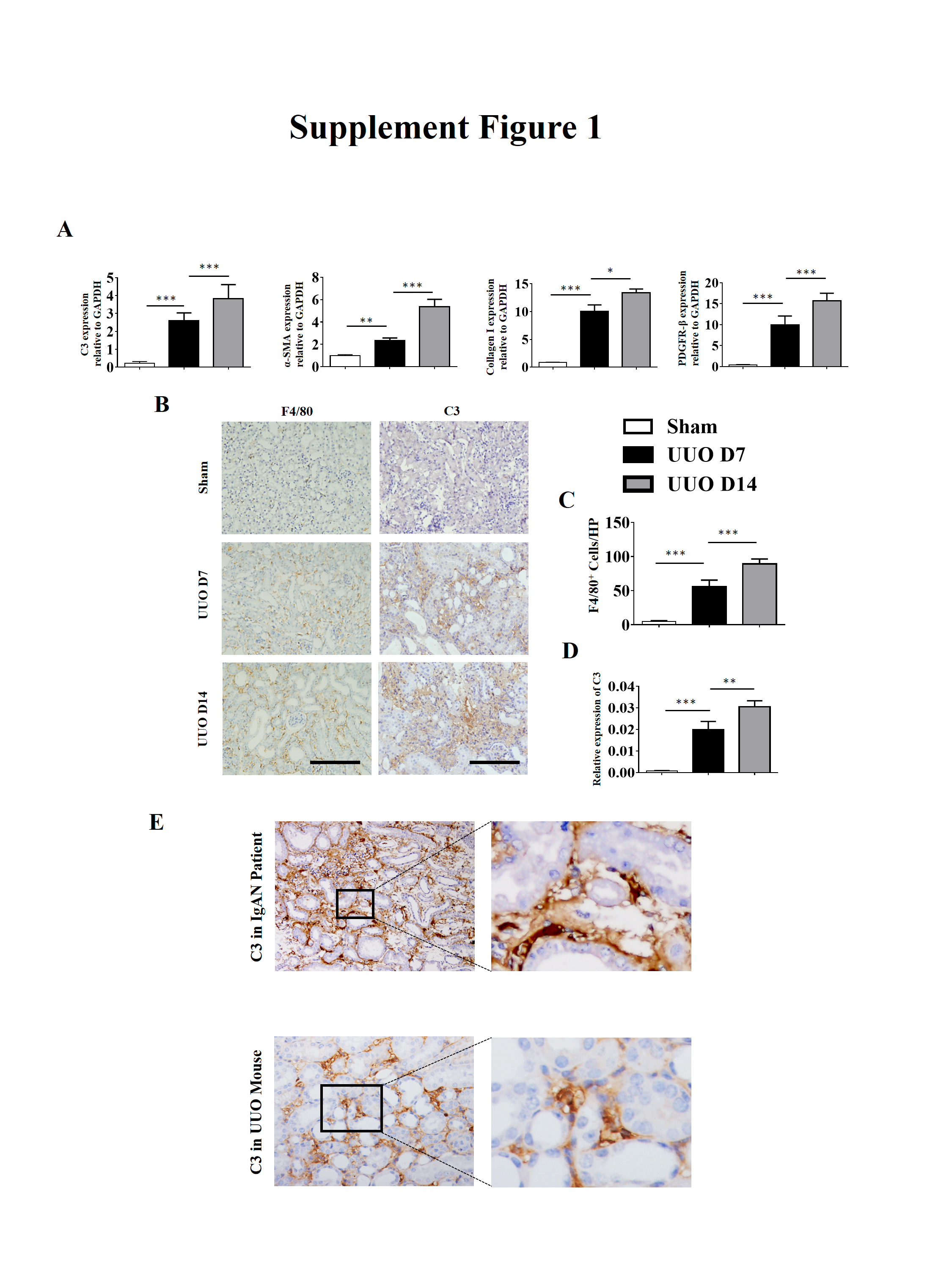

Supplement: Supplement Figure S1 — Complement C3 is increased in the obstructed kidney. (A) Real-time PCR showing relative renal mRNA levels of C3, fibrotic markers (α-SMA, PDGFR-β, and Collagen I) in sham control and UUO mice. (B) IHC staining showing F4/80 and C3 protein expression in the sham control and UUO mice; original magnification, ×400. Quantitative analysis of F4/80 (C) and C3 (D) positive cells were shown as mean ± SEM. n = 6 per group, Scale bar, 50 μm. (E) High magnification of C3 expression in IgA Patient and UUO mouse. The error bars represent the SEM. *P < 0.05; **P < 0.01; ***P < 0.001. [file Image_1.TIF]

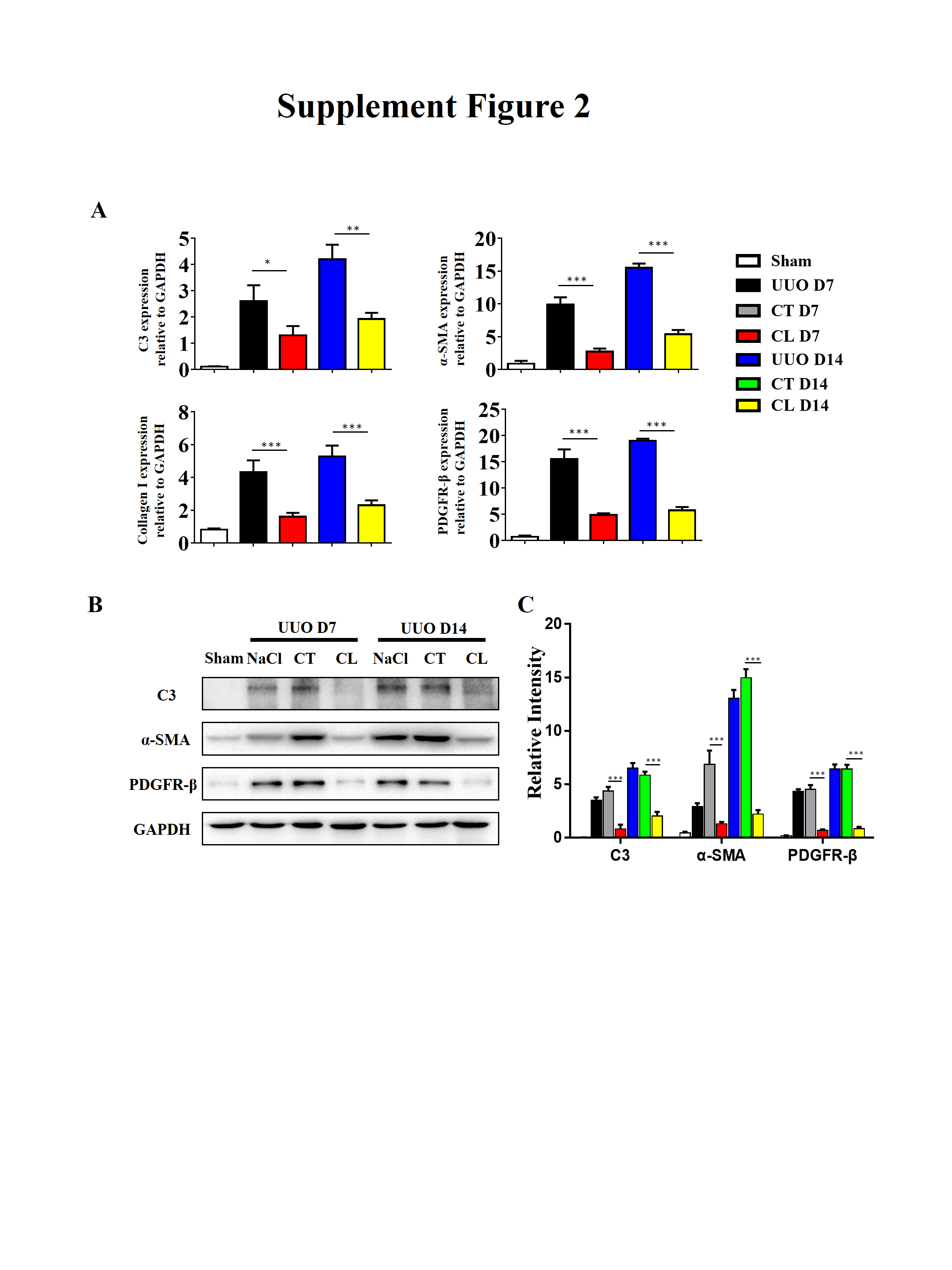

Supplement: Supplement Figure S2 — Macrophage depletion protects against renal fibrosis by inhibiting C3 expression. UUO mice were intravenously injected with clodronate liposomes and control liposomes. On days 7 and 14, mice were sacrificed, and the left kidneys were collected. The expression levels of C3 and fibrotic markers (α-SMA, PDGFR-β, and Collagen I) were detected by real-time PCR (A) and Western blot analyses (B), respectively. (C)The histogram shows the relative intensity for each marker normalized to GAPDH. n = 6 per group. The error bars represent the SEM. *P < 0.05; **P < 0.01; ***P < 0.001. [file Image_2.TIF]

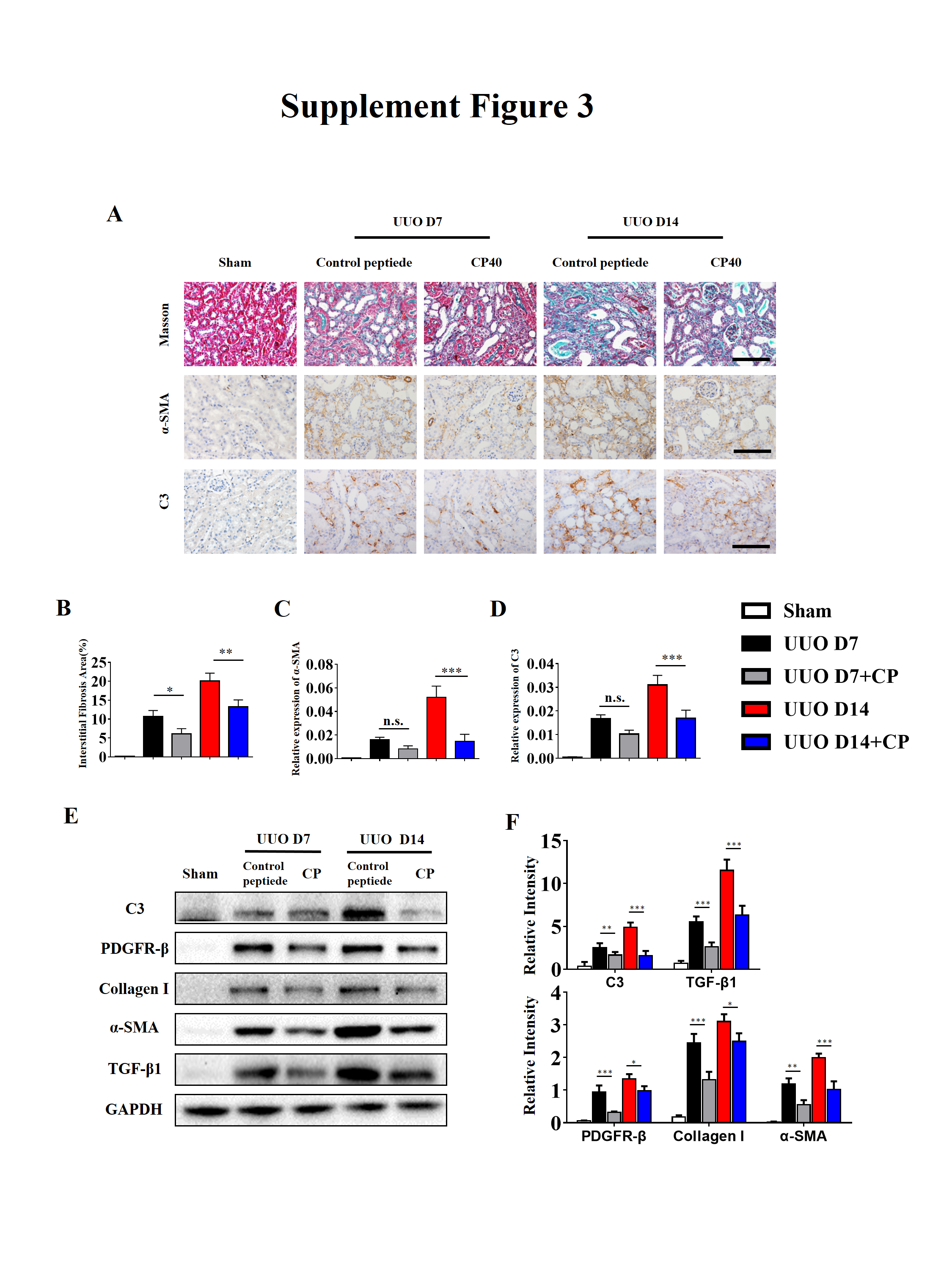

Supplement: Supplement Figure S3 — C3 deficiency reduces C3 expression and fibrosis during UUO. UUO mice were subcutaneously injected with control peptide and Cp40. On days 7 and 14, mice were sacrificed, and the left kidneys were collected. (A) Masson's trichrome staining indicates collagen deposition. IHC staining showing α-SMA and C3 protein expression in these groups (n = 6); original magnification, ×400. Scale bar, 50 μm. Quantitative analysis of interstitial fibrosis (B), a-SMA(C) and C3(D) positive cells were shown as mean ± SEM. (E) The expression levels of C3, TGF-β1, and fibrotic markers (α-SMA, PDGFR-β, and Collagen I) were detected by Western blot. (F) The histogram shows the relative intensity for each marker normalized to GAPDH. n = 6 per group. The error bars represent the SEM. *P < 0.05; **P < 0.01; ***P < 0.001. [file Image_3.TIF]

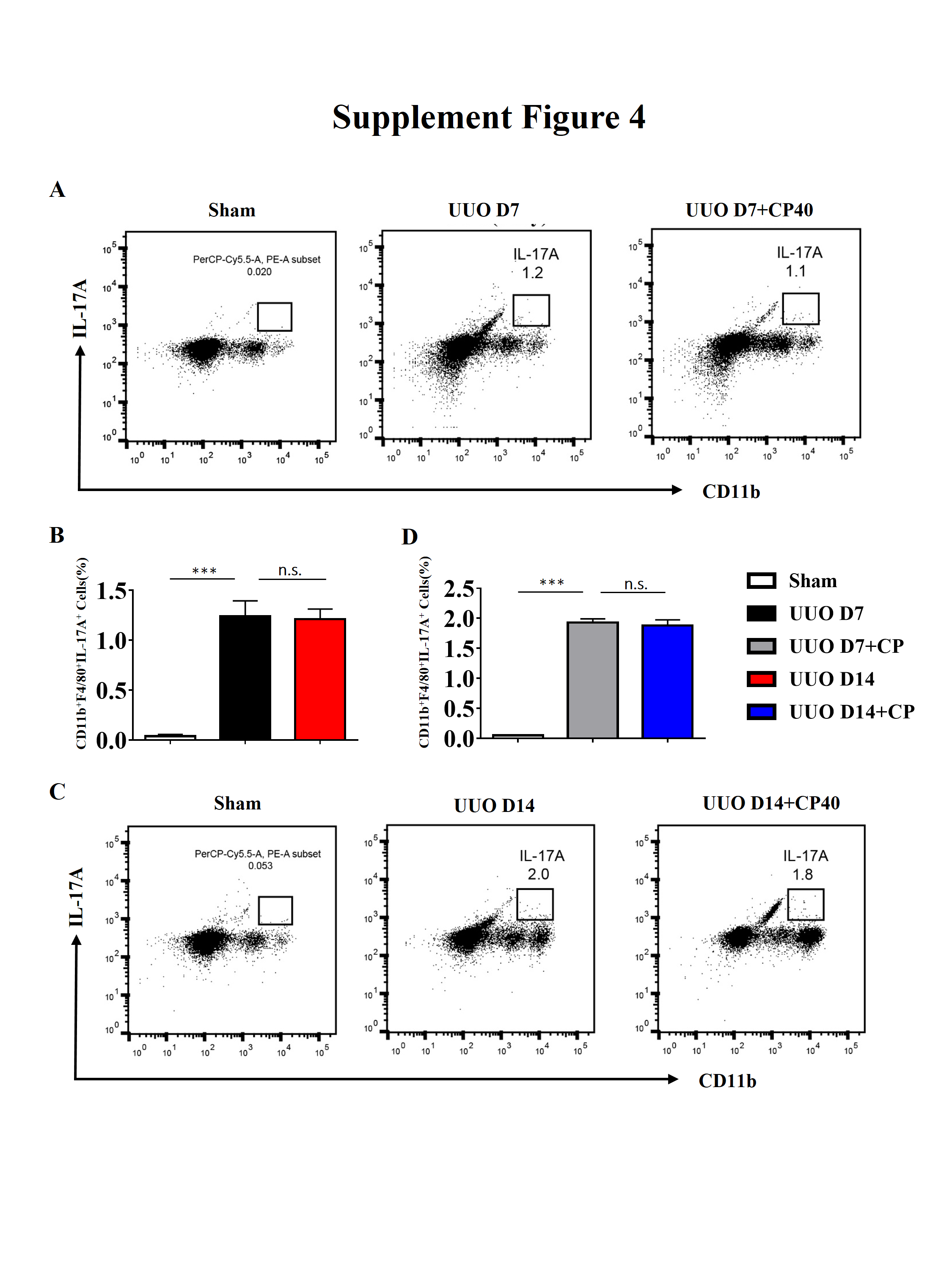

Supplement: Supplement Figure S4 — CD11b+F4/80+macrophages are not the main producer of IL-17A in kidney of UUO mice. Flow cytometric analysis of kidney cell suspensions from the obstructed kidneys injected with or without Cp40 at (A) 7 days and (C) 14 days post UUO; n = 6. Cells were stimulated in vitro with PMA/Ionomycin/Golgi-plug for 4 h. Specific staining of cell markers (anti-F4/80, anti-CD11b) and intracellular staining for IL-17A were performed. The F4/80+ cells were gated. Among them, the CD11b+IL-17+cells were further gated for the analysis. Plots are gated for live CD11b+F4/80+ IL-17A+ macrophages; numbers indicate events in the quadrants as percentages of all gated events. (B,D) Quantifications of CD11b+F4/80+ IL-17A+ cells as percentages of all kidney cells isolated from the C3 blockade UUO mice and UUO mice. The error bars represent the SEM. ***P < 0.001. The data were pooled from three independent experiments. [file Image_4.TIF]

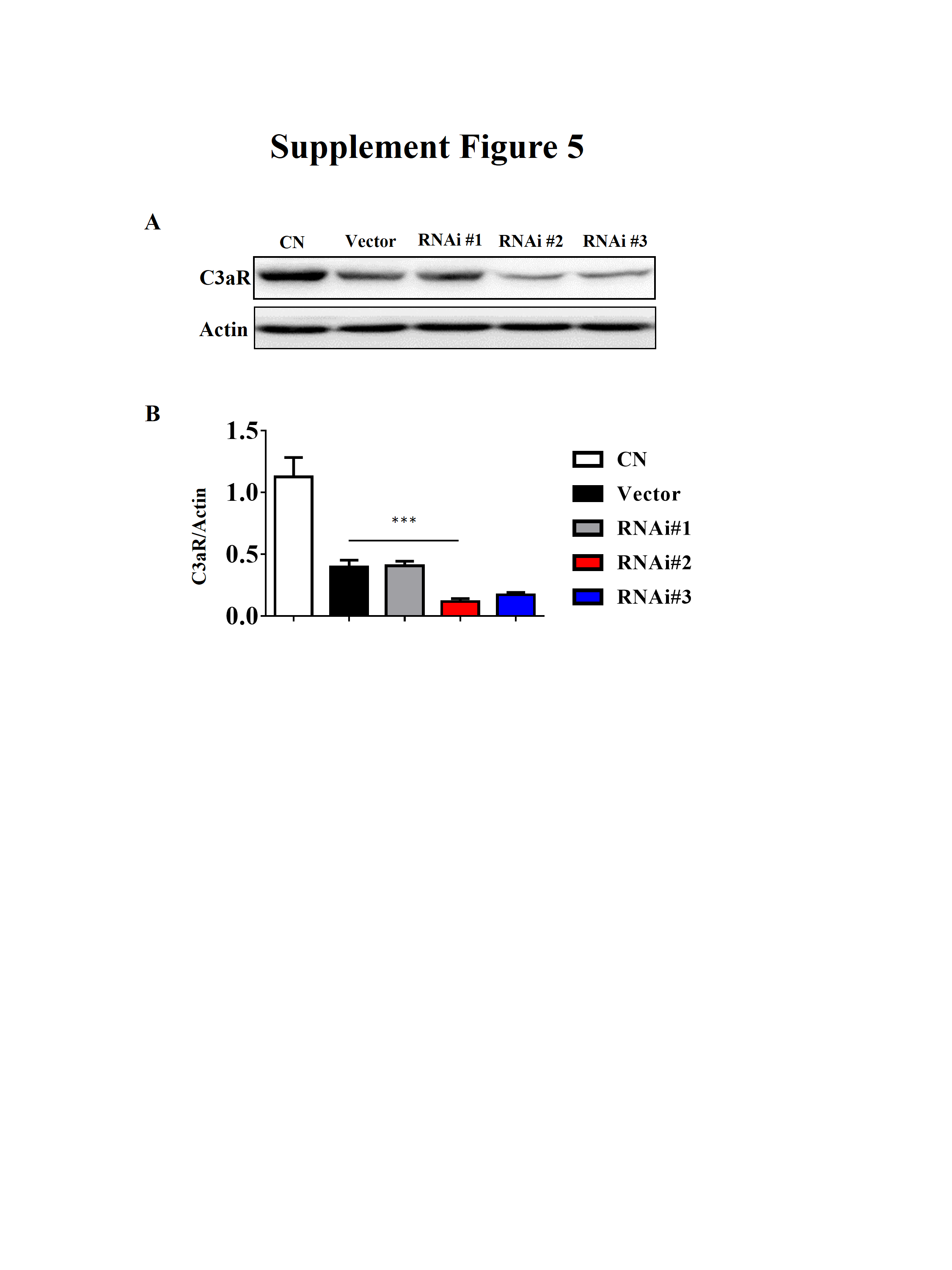

Supplement: Supplement Figure S5 — T cells were knocked down endogenous C3aR by using three small-interfering RNAs (siRNAs). (A) The expression levels of C3aR was detected by Western blot. (B) The histogram shows the relative intensity for each marker normalized to Actin. n = 3 per group. The error bars represent the SEM. ***P < 0.001. [file Image_5.TIF]
